# Adaptive NKG2C+ natural killer cells are related to exacerbations and nutritional abnormalities in COPD patients

**DOI:** 10.1186/s12931-020-1323-4

**Published:** 2020-03-04

**Authors:** Sergi Pascual-Guardia, Michelle Ataya, Isabel Ramírez-Martínez, José Yélamos, Roberto Chalela, Salomé Bellido, Miguel López-Botet, Joaquim Gea

**Affiliations:** 1 Respiratory Department, Hospital del Mar, Pg. Marítim 27, 08003 Barcelona, Spain; 2 Hospital del Mar Medical Research Institute (IMIM), Barcelona, Spain; 3 CIBERES, ISCIII, Barcelona, Spain; 4 Health and Experimental Sciences Department (CEXS), Universitat Pompeu Fabra, Barcelona, Spain; 5 Immunology Department, Hospital del Mar, Barcelona, Spain; 6 Psychiatry department, Hospital Torrecardenas, Almería, Spain; 7 Barcelona Respiratory Network, Barcelona, Spain

**Keywords:** Human Cytomegalovirus, NK cells, NKG2C, Chronic obstructive pulmonary disease, Exacerbation, Nutritional status, Fat free mas index

## Abstract

**Abstract:**

Chronic obstructive pulmonary disease (COPD) is a chronic and often progressive disorder with a heterogeneous presentation and frequent systemic manifestations. Several aspects like persistence in smoking habit, continuous exacerbations, alpha-1-antitrypsin deficiency and inflammatory-immune response, are involved in the pathophysiology and progression of the disease. However, the role of natural killer (NK) cells remains controversial. Otherwise, human cytomegalovirus (HCMV) infection has been reported to induce an adaptive differentiation and expansion of an NK cell subset which carries the CD94/NKG2C receptor, which may contribute to an upset immune defense. For these reasons, our objective is to assess the distribution of NK cells and their subset in COPD patients and some of its phenotypes.

**Methods:**

Peripheral blood samples were obtained from 66 COPD patients. HCMV serology and the proportions of total NK cells and the NKG2C+ and NKG2A+ subsets were evaluated by flow cytometry. The *NKG2C* genotype was also assessed.

**Results:**

Eighty-eight per cent of COPD patients were HCMV(+), and the proportions of total NK cells were higher in patients with severe-very severe airway obstruction than in those with only mild-moderate involvement. There were no differences in the proportions of NKG2C+ cells between controls and COPD, either among COPD patients classified by severity of the disease. However, the percentage of NKG2C+ cells were higher in COPD patients with frequent exacerbations than in occasional exacerbators, and higher in cases with reduced lean mass (Fat free mass index) than in those with normal nutritional status.

**Conclusion:**

These results suggest a relationship between levels of NKG2C+ cells in COPD patients and clinical variables closely linked to a poor/worse prognosis.

## Background

Chronic Obstructive Pulmonary Disease (COPD) is a highly prevalent entity which affects around 10% of the adult population in developed countries and entails significant social and healthcare costs [1]. It is characterized by a persistent airflow obstruction, although its clinical presentation is heterogeneous and includes systemic manifestations and frequent associations with a range of comorbidities [1]. Therefore, attempts have been made to further characterize or even personalize the diagnosis of COPD. Several phenotypes or/and endotypes of the disease have been described in recent years, including frequent exacerbators, the combination of COPD with bronchial asthma (ACO), a clear predominance of pulmonary emphysema or bronchiectasis, and associations with specific comorbidities or systemic manifestations such as cardiovascular involvement or nutritional abnormalities [2–4]. However, in the absence of suitable biomarkers, the diagnosis of these phenotypes is still essentially clinical. Identifying biomarkers would be helpful not only for diagnosis but for a better understanding of the pathophysiology of COPD and its heterogeneity and might contribute to the identification of novel therapeutic targets.

In COPD, the response to the insult (generally tobacco smoking) is fundamentally inflammatory at both pulmonary and systemic levels [5]. It is commonly accepted that neutrophils and T lymphocyte helper 1 (T_H_1) play a central role in the inflammatory response that characterizes this disorder [1, 6]. Other immune cells such as macrophages, regulatory T lymphocytes (Tregs) and natural killer (NK) cells have also been implicated in the pathophysiology of COPD [6, 7]. Some studies suggest that the inhibitory CD94 receptor is under expressed on NK cells from COPD patients, which may be related to an increase of granzyme B production [8, 9]. Although the specific role of NK cells has not been elucidated, it has been suggested that they are involved in the pathogenesis of pulmonary emphysema and in bronchial remodeling [10, 11].

For its part, human cytomegalovirus (HCMV) infection causes a highly prevalent, life-long persistent infection in between 40 and 90% of the general population [12]. Although it tends to be asymptomatic, this herpesvirus may have a severe pathogenic impact on congenital infections and in immunocompromised patients [13], and it is also associated with immune senescence and chronic inflammatory diseases such as atherosclerosis. To our knowledge, the prevalence of HCMV infection in patients with COPD has not been reported, but it is likely to mirror that of the general population, with increased prevalence rates of both entities in lower socioeconomic strata [1, 13]. After primary infection, HCMV remains latent, mainly in cells of myeloid lineage; it is occasionally reactivated, inducing the production of CD28_null_ T cells and promoting systemic inflammation in COPD patients [14], with a potential impact on disease progression. Also, alveolar macrophages in the lung may constitute an important viral reservoir [15] and CMV can be transmitted through secretions [16].

**Fig. 1:**
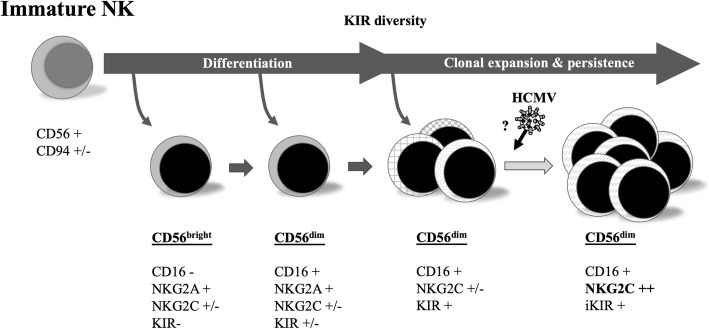
Differentiation and expansion of an NK cell subset, from immature NK to NKG2C++ (Currently accepted and adapted from M. López-Botet). Abbreviations: NK, natural killer cells; CD56, cluster of differentiation 56 or neural cell adhesion molecule, NK marker; CD94, cluster of differentiation 94 or NKG2, marker of NK cells and CD8^+^ T-lymphocytes; NKG2A, inhibitory NK cell receptor 2A; NKG2C, activating NK cell receptor; CD16, cluster of differentiation 16, marker of NK and other white cells; KIR, NK cell immunoglobulin-like receptors; iKIR, inhibitory KIR pattern

In addition to B cell production of HCMV-specific antibodies, T lymphocytes and NK cells play a fundamental role in the immune response to this pathogen [17]. In this context, HCMV has been reported to promote the specific differentiation and expansion of an NK cell subset hallmarked by expression of the CD94/NKG2C activating receptor specific for the HLA-E molecule, together with additional phenotypic and functional features, and which are currently known as adaptive NKG2C+ NK cells (Fig. 1) [16, 18]. This reconfiguration of the NK cell compartment is persistent, though its magnitude among infected individuals ranges from levels comparable to those detected in HCMV(−) subjects up to > 50% of total circulating NK cells. Studies in renal transplantation suggest that adaptive NKG2C+ NK cells may contribute to the immune defense against HCMV [19]. A homozygous deletion of the *NKG2C* gene has been reported, with frequencies of 6 and 8% in two Spanish cohorts of healthy donors [19, 20]. *NKG2C* gene copy number has been reported to influence the surface expression levels of the receptor [20]. It has been hypothesized that changes in the NK cell pool promoted by HCMV might influence the immune response to other infections, tumors or inflammatory-based diseases.

In this context, we considered a putative relation of adaptive NKG2C+ NK cell expansions with COPD phenotypes and systemic manifestations. The objective of this pilot study was to explore the levels of NK cells, and more specifically of the NKG2C+ subset, in COPD and in some of its most prevalent clinical forms.

## Methods

### Population

Sixty-six COPD patients were consecutively recruited from the outpatient clinic of the Respiratory Medicine Department at our institution. The diagnosis of COPD was made according to GOLD criteria [1]. Individuals with chronic alcoholism, bronchial asthma, neuromuscular diseases, neoplasms and, in general, entities and treatments that might alter per se the immune status were excluded from the study. In parallel, 13 healthy individuals of age and sex similar to those of the patients were also included.

### Procedures

Demographic and clinical data, including anthropometry, body composition, respiratory function and exercise capacity, were collected in all cases. In addition, peripheral blood samples were obtained for use in HCMV serology, as well as general analyses including a complete blood count, total NK cells and proportions of the cells expressing the activating receptor NKG2C or the inhibitory receptor NKG2A.

#### Clinical data

COPD was defined in the presence of a compatible clinical history and a post-bronchodilator FEV_1_/FVC ratio of < 70% [1]. The severity of airflow obstruction was defined by the FEV_1_ (% pred.) value, and patients were divided into those with mild-moderate (FEV_1_ > 50% pred.) and severe-very severe disease (FEV_1_ < 50% pred.). ‘Frequent exacerbators’ were defined as patients with two or more moderate to severe exacerbations (for which they contacted the health system) in the year prior to the study. Patients were also divided according to their nutritional status (see below), presence of peripheral blood eosinophilia (> 300 cells/mm^3^), presence of predominant emphysema (as assessed by computed tomography [CT]), or presence of bronchiectasis on CT. Dyspnea, both at baseline and at the end of submaximal exercise (see below), was assessed using the Borg scale [21]. In turn, dyspnea during activities of daily living was evaluated using the modified Medical Research Council (MRC) scale [22].

#### Respiratory function and exercise tolerance

Forced spirometry with a bronchodilator test, as well as determination of static lung volumes (plethysmography) and carbon monoxide transfer coefficient (DL_CO_) were performed according to standardized procedures, and values are expressed as percentages of reference for a Mediterranean population [23–25]. A 6-min walking test was also performed, measuring distance and symptoms and monitoring oxyhemoglobin and heart rate (pulse oximeter 9590 Onyx Vantage, Nonin Medical Inc., Plymouth, MN). The test was carried out at least twice and the result with the highest distance was chosen.

#### Nutritional status

Anthropometric variables were calculated, and body composition was determined through bioelectrical impedance (Bodystat 1500, Bodystat Ltd., Isle of Man, UK). Patients were then divided into those with nutritional abnormalities (Body mass index [BMI] thresholds < 20 kg/m^2^ or alternatively < 18.5 kg/m^2^) or those with normal nutritional status [5, 26]. Moreover, regarding body composition patients were also divided in those with a reduced lean mass (limits for fat free mass index [FFMI] < 18 kg/m^2^ in men and < 15 kg/m^2^ in women; or alternatively, < 16 kg/m^2^ in men and < 14.5 kg/m^2^ in women) [5].

#### Emphysema according to computed tomography

Two expert radiologists independently assessed the CT, classifying patients into those with predominant or non-predominant emphysema and those with or without bronchiectasis. The few patients in whom discrepancies were recorded were excluded from the study.

#### NK cell markers and *NKG2C* genotype (flow cytometry)

**Fig. 2:**
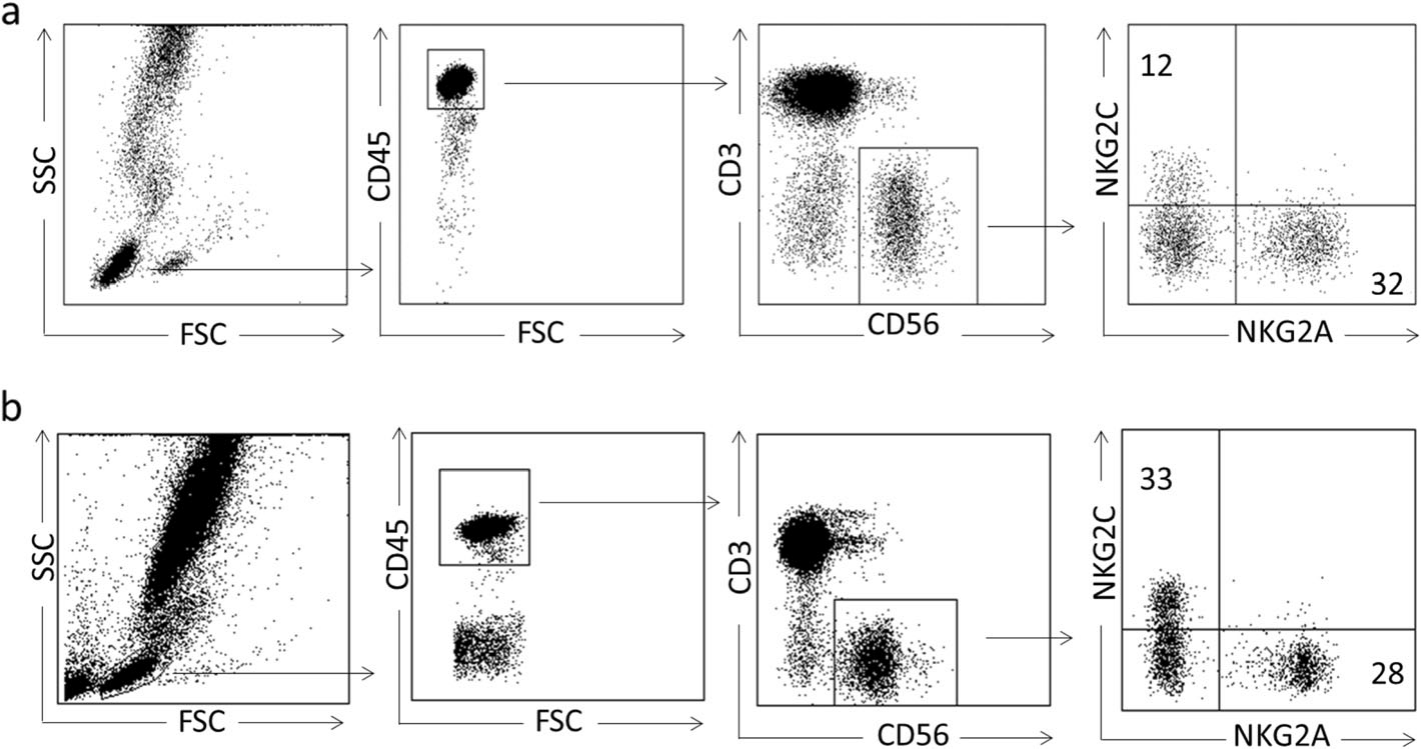
Flow cytometry gating strategy to define NK cell subsets in two representative patients. In Fig. 2**a** we show a patient with a low percentage (< 20%) of NKG2C and in Fig. 2**b** we show a patient with a high percentage (> 20%) of them

This analysis was performed by flow cytometry with fresh blood samples drawn in tubes with EDTA and handled according to the procedure described elsewhere [27]. Briefly, the sample was pretreated with saturating concentrations of human aggregated immunoglobulins, in order to block FcγR. Labeling was carried out with various combinations of antibodies in order to identify NK cell subsets. The following antibodies were used for direct immunofluorescence: APC-H7-conjugated anti-CD3, PerCP-conjugated anti-CD45, FITC-conjugated anti-CD56 (BD Biosciences, San Diego, CA). NK cells were identified as CD45 + CD3-CD56+ lymphocytes, and subsets were identified with antibodies APC-conjugated anti-NKG2C (R & D systems, Minneapolis, MN), and PE-conjugated anti-NKG2A (Beckman Coulter, Brea, CA). After washing and erythrocyte lysis, samples were analyzed with a FACS Canto cytometer. Data were processed with DIVA software (BD Biosciences), and absolute and relative counts of NK cells (related to total lymphocytes) and of each of the NK subsets (in this case in relation to total NK) were obtained [18] (Fig. 2). *NKG2C* zygosity was assessed as previously described [28].

### Statistical analysis

The sample size for the study was calculated accepting an α risk of 0.05 and a β risk of less than 0.2 in a bilateral contrast. This meant that a minimum of 55 patients was required to detect a difference of 0.09 units or more. It was assumed that the proportion would be 0.05 in the group of preference, estimating the absence of losses, given the characteristics of the study.

The normality of the quantitative variables was assessed using the Shapiro-Wilk test. The quantitative variables with a normal distribution are expressed as means ± standard deviation (x ± SD) and those with a non-normal distribution as median (interquartile range) (x̅[IR]). The qualitative variables are showed as percentages. The comparisons between groups were carried out using the t-test for paired data or the Mann-Whitney U test as appropriate, or the X^2^ test in dichotomous variables. In the multivariate analysis we included those variables that were significant in the univariate analysis, as well as those clinically relevant or with a *P* value < 0.2. Regarding the NKG2C+ cell count, two groups were differentiated, according to the threshold of 20% which was chosen as a function of the x value of these cells observed in healthy seronegative subjects plus two SD [18, 29]. Correlations between variables were assessed using Pearson or Spearman coefficients, according to the distribution of the variables. All statistical analyses were performed using SPSS 24.0 (SPSS Inc., Chicago, IL, USA), and a *p*-value < 0.05 was considered statistically significant.

## Results

Clinical characteristics of COPD patients are shown in Table 1. Most of these patients had a normal nutritional status (BMI 26.2 ± 6.5 kg/m^2^), although their lean mass decreased (only 65% exhibited normal body composition using the FFMI). Regarding lung function, up to 76% of patients showed severe to very severe airflow obstruction (FEV_1_ < 50% pred.). In COPD patients, 26% had peripheral blood eosinophilia > 300/ul and the serology for HCMV was positive in 87.9%, similar to the control group (81.8%) and to previous reports in the general population of comparable age [30], We did not find significant relationship between HCMV+ serology and NK and NKG2C+ cells (*p* = 0.1 and 0.5 respectively).

**Table 1:** Main general and functional data of the overall population of COPD patients

COPD patients	n	$\overline{\mathrm{X}}$±SD
Age (years)	66	70 ± 8
Sex (male %)	66	52(82)
Tobacco (pack-year)	66	41 ± 11
**Anthropometry**		
Weight (kg)	66	71 ± 18
BMI (kg/m^2^)	66	26.2 ± 6.5
FFMI (kg/m^2^)	52	16.9 ± 2.7
Exacerbations (n)	66	1.3 ± 1.2
**Blood test and flow cytometry**		
Eosinophils (total/mm^3^)	66	199 ± 164
HCMV+ (%)	35	31(89)
NK	59	19.1 ± 10
NKG2C+	63	14.4 ± 17
NKG2A+	46	29.2 ± 17
**Functional testing**		
FEV_1_ (% pred.) pre/post bd	66	37 ± 14/39 ± 15
FEV_1_/FVC (%) pre/post bd	66	44 ± 11/44 ± 12
RV/TLC (%)	60	63 ± 11
DLco (% pred.)	60	44 ± 20
Kco (% pred.)	60	51 ± 20
SpO_2_ (%)	66	93 ± 3
6MWT (m)	56	359 ± 122
**CT Scan**		
Emphysema (%)	58	44(76)
Bronchiectasis (%)	58	13(22)

Data are presented as means ± standard deviation ($\overline{\mathrm{X}}$ ±SD) or n(percentages). Abbreviations: *BMI* body mass index; *FFMI* fat free mass index; *FEV*_*1*_ forced expiratory volume in the first second; *bd* bronchodilator; *FVC* forced vital capacity; *RV* residual volume; *TLC* total lung capacity; *DLco* carbon monoxide transfer coefficient; *Kco* Krogh index (DLco/alveolar volume; *SpO*_*2*_ oxygen saturation (pulse oximetry); *6MWT* six-minute walking test distance. *CT* computed tomography,

### NK cells

**Fig. 3:**
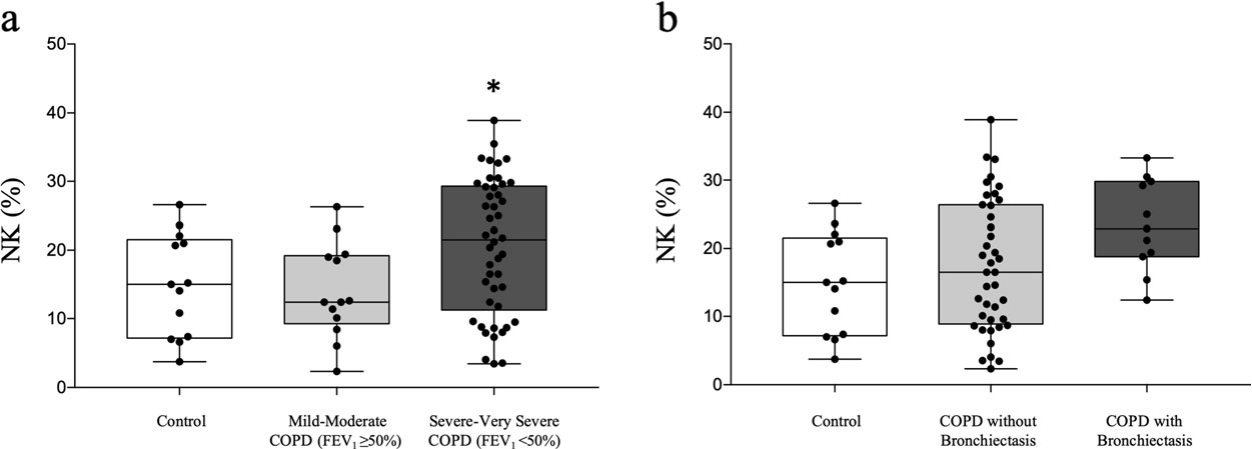
Percentages of NK cells in (**a**) control vs. severe-to-very severe vs. mild-to-moderate COPD patients; (**b**) control vs. COPD patients with and without bronchiectasis. * *p* value < 0.05

The proportions and absolute numbers of CD56+ CD3- NK cells were similar between controls and COPD patients. Analysing subpopulations of COPD patients, the proportions of NK cells were also higher in the group with severe-very severe disease than in those with mild-moderate disease (20.6 ± 9.8% vs. 13.9 ± 6.9%, *p* < 0.05) (Fig. 3a). COPD patients with bronchiectasis in CT displayed higher proportions of NK cells than patients without this abnormality (23.4 ± 6.7% vs 17.3 ± 9.7%, *p* < 0.05) (Fig. 3b). Interestingly, proportions of total NK cells did not differ significantly between active smokers and ex-smokers (15.8 ± 7.9 vs 20.1 ± 9.9, *p* = 0.09). Neither the patients with FFMI> 18 kg/m^2^ [men] and > 15 kg/m^2^ [women] showed different levels of total NK cells (572 ± 342 vs 340 ± 221, *p* = 0.07). Predominant emphysema (defined either by CT or a decreased DLco value) or eosinophilia appeared unrelated to the proportion or total NK cells. By contrast, the proportion of NK cells was inversely correlated to the FVC (r = − 0.358, *p* < 0.01), but not differ significantly with TLC (*p* = 0.09), FEV_1_ (*p* = 0.08) and the distance obtained in the 6-min walking test (*p* = 0.09).

### NKG2C+ and NKG2A+ cells

**Fig. 4:**
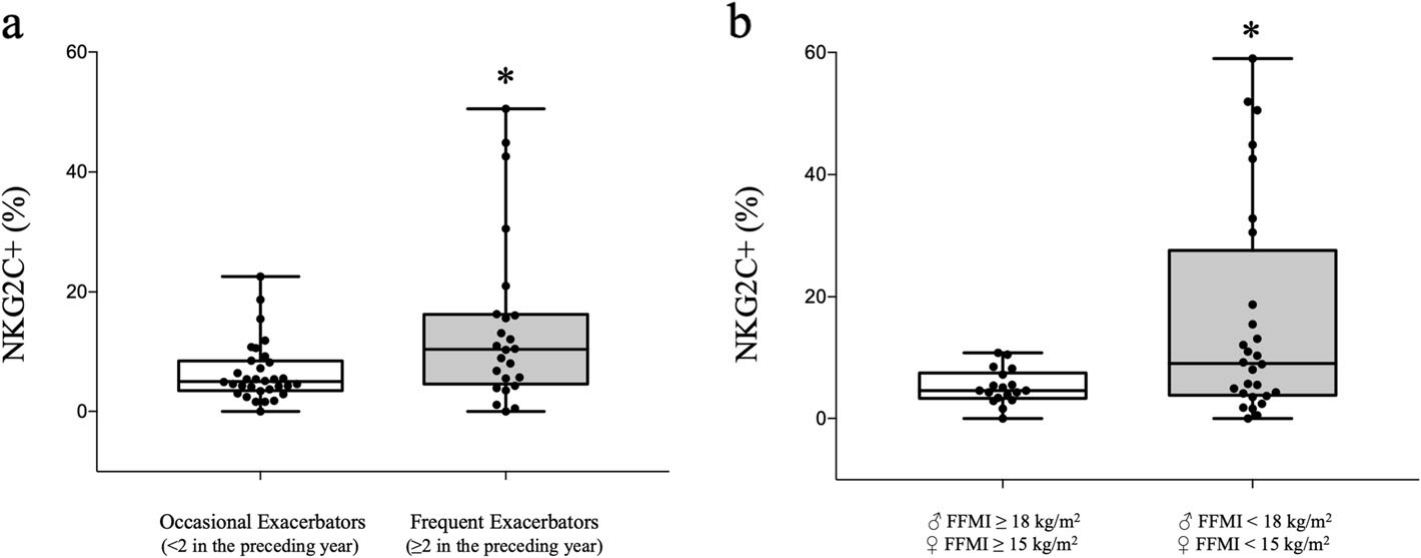
Percentages of NKG2C+ cells in: (**a**) occasional vs. frequent exacerbators; (**b**) normal body composition vs. low FFMI (< 18 kg/m^2^ [♂] & < 15 kg/m^2^ [♀]). * *p* value < 0.05

Although the proportions of NKG2C+ cells were similar in controls and COPD patients (13.9 ± 8.0 vs 14.4 ± 17.1, *p* = 0.9), higher values were observed in frequent than in occasional exacerbators (19.6 ± 20% vs. 10.5 ± 13, *p* < 0.05), albeit with a substantial dispersion of values (Fig. 4a). In addition, patients with a reduced FFMI (< 18 kg/m^2^ [men] and < 15 kg/m^2^ [women]) showed higher NKG2C+ cell proportions than patients with conserved body composition (19.4 ± 21% vs. 8.1 ± 13%, *p* < 0.05, respectively) (Fig. 4b). Unfortunately, the low number of patients with BMI < 18.5 kg/m^2^ or FFMI< 16 kg/m^2^ (men) and < 14.5 kg/m^2^ (women) did not allow comparisons using those alternative limits. No differences were observed between COPD groups regarding the severity of the disease, smoking status, predominance of emphysema or presence of bronchiectasis. Finally, a positive correlation was observed between NKG2C+ cells and basal SpO_2_ (r = 0.335, *p* < 0.01) and a trend towards a positive correlation with the number of exacerbations (r = 0.229, *p* = 0.07). The proportions of NKG2A+ cells were lower in COPD patients than in controls (29.2 ± 17% vs 48.2 ± 18 respectively, *p* < 0.05). No differences were observed between COPD groups regarding the severity of the disease, nutritional or body composition abnormalities, number of exacerbations, smoking status, number of eosinophils or predominance of emphysema/bronchiectasis. Nor were significant correlations found between the different variables and the levels of NKG2A+ cells. *NKG2C* gene deletion was detected in 24.3% of the patients (being homozygous in 2.4% and hemizygous in the rest), similar to previous reports in the general population [18]. Interestingly, patients with deletions had greater air trapping than those without this functional abnormality (RV/TLC 67 ± 7 vs. 59 ± 11%, *p* < 0.05 respectively), and were similar for the rest of variables.

### NKG2C+ threshold 20%

In a complementary approach, when patients were stratified according to their % of NKG2C+ NK cells with respect to the threshold (> 20%) described in statistical analysis section, those with higher percentages showed poorer body composition (low FFMI) and more exacerbations (Table 2). Moreover, the proportions of frequent exacerbators, or patients with either reduced BMI or FFMI, were increased among cases with NKG2C+ cells > 20%; conversely, the proportions of subjects with predominant emphysema (CT) or peripheral eosinophilia were reduced in this group.

**Table 2:** Main general, functional and flow cytometric data of groups of COPD patients

	n	NKG2C+ < 20%	NKG2C+ > 20%	Significance
Age (years)	66	71 ± 8	67 ± 9	ns
Sex male n(%)	66	43(81)	11(85)	ns
Tobacco (pack-year)	66	42 ± 11	38 ± 11	ns
**Anthropometry**				
Weight (kg)	66	72 ± 19	69 ± 16	ns
BMI (kg/m^2^)	66	26.5 ± 6.5	24.7 ± 6.5	ns
BMI < 20 n(%)		7(13)	5(38)	*p* < 0.001
FFMI (kg/m^2^)	52	17.2 ± 2.5	15.7 ± 1.7	*p* = 0.05
Low FFMI < 18♂ & < 15♀ n(%)		24(57)	9(90)	p < 0.001
**History of exacerbations**				
Exacerbations (n)	66	1.21 ± 1.2	1.85 ± 0.9	*p* < 0.05
Frequent exacerbators (%)		19(36)	8(62)	*p* < 0.001
**Blood test and flow cytometry**				
Eosinophils (total/mm^3^)	66	206 ± 176	173 ± 104	ns
Eosinophils > 300/mm^3^ (%)		15(28)	2(15)	*p* < 0.05
HCMV+ (%)	35	28(88)	3(100)	ns
NK	59	18.7 ± 10	20.5 ± 8	ns
NKG2A+	46	30.1 ± 18	22.2 ± 9	ns
**Functional testing**				
FEV_1_ (% pred.) pre/post bd	66	37 ± 13/39 ± 14	37 ± 18/38 ± 21	ns
FEV_1_/FVC (%) pre/post bd	66	44 ± 11/44 ± 11	45 ± 13/46 ± 14	ns
RV/TLC (%)	60	63 ± 10	63 ± 16	ns
DLco (% pred.)	60	45 ± 18	43 ± 29	ns
Kco (% pred.)	60	52 ± 20	49 ± 24	ns
SpO_2_ (%)	66	93 ± 3	92 ± 4	ns
6MWT (m)	56	362 ± 118	350 ± 141	ns
**CT Scan**				
Emphysema (%)	58	37(80)	7(58)	*p* < 0.01
Bronchiectasis (%)	58	10(22)	3(25)	ns

Data are represented as means ± standard deviation or n(percentages). Abbreviations: *BMI* body mass index; *FFMI* fat free mass index; *HCMV* human cytomegalovirus; *NK* Natural Killer cells; *FEV*_*1*_ forced expiratory volume in the first second; *bd* bronchodilator; *FVC* forced vital capacity; *RV* residual volume; *TLC* total lung capacity; *DLco* carbon monoxide transfer coefficient; *Kco* Krogh index (DLco/alveolar volume; *SpO*_*2*_ oxygen saturation (pulse oximetry); *6MWT* six-minute walking test distance; *CT* computed tomography,

When controlling for the covariates age, sex, FEV_1_% and the FFMI, only the number of exacerbations emerged as an independent risk factor associated with high levels of NKG2C+ (OR 3 [1.1–8.4], *p* < 0.05).

## Discussion

This is the first study to analyse the presence of NKG2C+ cells in patients with COPD. Although the adaptive response of NK cells to HCMV infection varies widely in healthy subjects, the most interesting findings were that patients with COPD and frequent exacerbations or with nutritional abnormalities (lower FFMI) showed increased proportions of NKG2C+ cells.

The search for blood biomarkers to improve the diagnosis of the different COPD phenotypes is one of the priorities in respiratory research. On the one hand, their use would facilitate the clinical management of these patients by improving their selection for more expensive and complex interventions, and would help monitor their response to therapy; on the other, biomarkers may contribute to identifying additional mechanistic clues underlying the development of the disease, possibly leading to the design of novel therapeutic approaches. Thus far, the vast majority of markers described for the disease, its complications, comorbidities and systemic manifestations are unspecific, focusing fundamentally on inflammation, oxidative stress and detection of products derived from the lung, endothelium, or muscle structure or metabolism. Our data suggest that the adaptive response of NK cells to HCMV may be related to a greater predisposition to exacerbation in patients with COPD. Although further studies are still needed, titration of the immune response could help clinicians as a biomarker of the disease in these patients.

NK cells are innate lymphoid cells which contribute to the elimination of infected and neoplastic cells, regulating the development of adaptive humoral and cell-mediated responses [31]. NK cells display different surface receptors, which regulate their effector functions (i.e., cytotoxicity and cytokine production). Various NK cell subsets have been defined according to the surface expression of these molecules. Among them, the homologous inhibitory CD94/NKG2A and activating CD94/NKG2C receptors specifically recognize HLA-E, a non-classical HLA class I molecule. It is well established that there is a persistent differentiation and expansion of NKG2C+ NK cells (albeit with varying magnitudes) in response to HCMV infection both in healthy individuals and in different pathological conditions [32, 33]. In our cohort of patients,

To date, studies of the numbers and functions of NK cells in COPD patients, both in blood and in the pulmonary compartment, are controversial. Some authors have reported that NK cells are reduced in the bronchoalveolar lavage (BAL) of subjects with chronic bronchitis [34]. However, others have observed raised numbers of NK cells with an increased cytotoxic function in the induced sputum of COPD patients [8, 35]. One possible explanation to these controversial data are the different origin of the sample. As regards NK cells in peripheral blood, some authors have found them to be elevated in patients with COPD [36], but others have reported normal or reduced proportions with either preserved or impaired function [8, 37–39]. Some of the discrepancy observed between lung and blood compartments may be attributed to the fact that the former are more closely related to COPD, and the latter appear to be influenced more by smoking [40]. However, we stress that in the present study we did not observe differences in NK cell percentages between active smokers and ex-smokers.

Regarding the specific role of NK cells in COPD, some authors have reported that their cytotoxic mediators (i.e., granzyme B and perforin) may be involved in inducing apoptosis in the lungs, thus facilitating emphysema [10, 11]. However, these results have not been confirmed by other authors [41]. It has been suggested that the enhanced cytotoxicity of NK cells against lung epithelial cells in COPD is mediated by dendritic cell transpresentation of IL-15 through the interleukin 15 receptor subunit α (IL-15Rα) [42]. To our knowledge, the only study to address the relationship between COPD severity and NK cells showed that lung tissue CD56+ lymphocytes (including both NK and a T cell subset) increased their cytotoxicity against lung parenchyma cells and may contribute to emphysema progression [43]. In our cohort we did not observe higher numbers of NK cells in the patients with predominant emphysema, but we did observe higher numbers of NK cells in patients with COPD and bronchiectasis, a finding that has also been reported previously [44, 45]. The progressive lung damage resulting from a ‘vicious cycle’ of recurrent exacerbations and a poorly regulated inflammatory response has been proposed as one of the main pathogenic mechanisms of bronchiectasis [46].

As we noted above, there is some evidence of a relevant role for NK cells in the exacerbations of the disease [47]. In fact, a differential expression of genes related to NK cell activity was detected in COPD patients suffering exacerbations, and this expression profile appeared related to the number of these acute episodes [48]. Furthermore, the immunomodulatory role that NK cells seem to play in COPD has led some authors to propose the development of new therapeutic strategies for this pulmonary disease [8, 49]. For instance, blockade of CD137 expression has been reported to downregulate in vitro proinflammatory cytokines and granzyme B expression in CD8+ T and NK cells from COPD patients [50].

As for NK cell receptors, it has been reported that cytotoxic cells (NK and or CD8+ T cells) expressing NKG2D (another NK activating receptor) are increased in the BAL of both smokers and COPD patients [51]. In fact, some years ago our group reported that tobacco causes a persistent expression of the NKG2D ligand in the bronchial epithelium, which may be involved in the development of lung disease [52]. Moreover, the expression of NKG2D induced by tobacco appears to modulate the response of NK cells to infections [53]. Regarding peripheral blood, increases in receptors have occasionally been reported in COPD patients [36], although other authors have been unable to find such changes [37]. Yet, steroids decreased in vitro the expression of NKG2D in NK cells [37]. To our knowledge, no information has been published on NKG2C+ cells in COPD.

Our results reinforce the notion that the proportions of NK cells are higher in peripheral blood of COPD with more advanced disease, the presence of bronchiectasis, and a greater number of exacerbations. On the other hand, the proportions of NK cells that expressed the NKG2C receptor appear to be higher in frequent exacerbators and in patients with nutritional abnormalities. In fact, factors such as the distribution of different NK cell subsets, disease severity, the number of previous exacerbations and nutritional status may partially explain the discrepancies observed in previous studies conducted in COPD.

Several different interpretations may be proposed to explain the differences in the NK cell compartment profile features observed in COPD patient subpopulations. The increase in total NK cells may be related to the presence of persistent inflammation linked to the disease itself, especially at the more advanced stages. On the other hand, we could speculate that the increase in the NKG2C+ population could reflect a response to recurrent HCMV reactivation, promoted by repeated exacerbations and/or poor nutritional status. In this regard, it is interesting that proinflammatory cytokines (e.g., TNFα) were reported to promote reactivation of HCMV replication in myelomonocytic cells [54–56]. A prospective study of the presence of the virus in blood and saliva or respiratory secretions is needed to address this possibility. Alternatively, one might speculate that individuals displaying higher NKG2C+ expression levels would be susceptible to experience a worse progression of COPD. It should be noted that NKG2C+ NK cells efficiently mediate cytotoxicity and secrete proinflammatory cytokines (i.e., IFN-γ and TNF-α), particularly on antibody-dependent activation through the FcγR-IIIA (CD16) receptor. It is known that these proinflammatory cytokines are important mediators of weight loss, which is frequently associated with chronic diseases such as COPD [5]. To address this causal hypothesis would require a longitudinal study with serial determinations of NKG2C+ cells in patients ideally recruited in early stages of the lung disease.

This pilot study has several limitations that should be mentioned. First, the number of control subjects was low. Nevertheless, our objective was to evaluate the distribution of the NK cells, and more specifically of the NKG2C+ subset, in COPD and in some of its most prevalent clinical forms. Moreover, the distribution of NK and NKG2C+ cells in control subjects has been described elsewhere in the literature [16, 20]. Second, the distribution of the COPD patients stratified according to their NKG2C+ threshold of 20% was unequal (53 vs 13) and with a high standard deviation. As far as we know, this is the first study to evaluate the distribution of NKG2C + cells in these patients, and so the distribution could not be predicted. As previously mentioned, the threshold of 20% was chosen as a function of the x value of these cells observed in healthy seronegative subjects plus two SD. Tirth, due to technical issues, we don’t have all the data in all patients. Despite this lack of information, the power of statistical analysis is sufficient to detect the aforementioned differences.

## Conclusions

The present study indicates that total NK cells and the NKG2C+ subset may constitute markers suitable for assessing factors underlying COPD heterogeneity in greater depth.

## Data Availability

Authors don’t have Institutional Review Board’s approval to share data. In the next studies we will add a line in informed consent forms that stated the data would be shared publicly.

## Abbreviations

ACO: Asthma COPD Overlap
BAL: Bronchoalveolar Lavage
BMI: Body Mass Index
CD: Cluster of Differentiation
COPD: Chronic Obstructive Pulmonary Disease
CT: Computed Tomography
DL_CO_: Carbon monoxide transfer coefficient
EDTA: Ethylenediaminetetraacetic Acid
FEV_1_: Forced Expiratory Volume in the first second
FFMI: Fat Free Mass Index
FVC: Forced Vital Capacity
GOLD: Global initiative for chronic Obstructive Lung Disease
HCMV: Human Cytomegalovirus
HLA: Human leukocyte antigen
IFN-γ: Interferon gamma
IL: Interleukin
IR: Interquartile Range
MRC: Medical Research Council
NK: Natural Killer
OR: Odds Ratio
RV: Residual Volume
SD: Standard deviation
TLC: Total Lung Capacity
TNF-α: Tumor Necrosis Factor alpha

## References

[1] Vogelmeier CF, Criner GJ, Martinez FJ, Anzueto A, Barnes PJ, Bourbeau J (2017). Erratum to "global strategy for the diagnosis, management, and prevention of chronic obstructive lung disease 2017 report: GOLD executive summary" [arch Bronconeumol. 2017;53:128-49]. Arch Bronconeumol.

[2] Miravitlles M, Soler-Cataluna JJ, Calle M, Molina J, Almagro P, Quintano JA (2017). Spanish guidelines for Management of Chronic Obstructive Pulmonary Disease (GesEPOC) 2017. Pharmacological treatment of stable phase. Arch Bronconeumol.

[3] Garcia-Aymerich J, Gomez FP, Benet M, Farrero E, Basagana X, Gayete A (2011). Identification and prospective validation of clinically relevant chronic obstructive pulmonary disease (COPD) subtypes. Thorax..

[4] de-Torres JP, Marin JM (2017). Differences between GesEPOC and GOLD in 2017. Arch Bronconeumol.

[5] Gea J, Sancho-Munoz A, Chalela R (2018). Nutritional status and muscle dysfunction in chronic respiratory diseases: stable phase versus acute exacerbations. J Thorac Dis.

[6] Barnes PJ (2008). Immunology of asthma and chronic obstructive pulmonary disease. Nat Rev Immunol.

[7] Fairclough L, Urbanowicz RA, Corne J, Lamb JR (2008). Killer cells in chronic obstructive pulmonary disease. Clin Sci (Lond).

[8] Hodge G, Mukaro V, Holmes M, Reynolds PN, Hodge S (2013). Enhanced cytotoxic function of natural killer and natural killer T-like cells associated with decreased CD94 (Kp43) in the chronic obstructive pulmonary disease airway. Respirology..

[9] Cong J, Wei H (2019). Natural killer cells in the lungs. Front Immunol.

[10] Hodge S, Hodge G, Nairn J, Holmes M, Reynolds PN (2006). Increased airway granzyme b and perforin in current and ex-smoking COPD subjects. COPD..

[11] Suzuki M, Sze MA, Campbell JD, Brothers JF, Lenburg ME, McDonough JE (2017). The cellular and molecular determinants of emphysematous destruction in COPD. Sci Rep.

[12] Astegiano S, Sidoti F, Costa C, Ostorero A, Alotto D, Castagnoli C (2010). Human cytomegalovirus load in fresh and glycerolized skin grafts. New Microbiol.

[13] Crough T, Khanna R (2009). Immunobiology of human cytomegalovirus: from bench to bedside. Clin Microbiol Rev.

[14] Tan DB, Amran FS, Teo TH, Price P, Moodley YP (2016). Levels of CMV-reactive antibodies correlate with the induction of CD28(null) T cells and systemic inflammation in chronic obstructive pulmonary disease (COPD). Cell Mol Immunol.

[15] Poole E, Juss JK, Krishna B, Herre J, Chilvers ER, Sinclair J (2015). Alveolar macrophages isolated directly from human Cytomegalovirus (HCMV)-seropositive individuals are sites of HCMV reactivation in vivo. J Infect Dis.

[16] Lopez-Botet M, Muntasell A, Vilches C (2014). The CD94/NKG2C+ NK-cell subset on the edge of innate and adaptive immunity to human cytomegalovirus infection. Semin Immunol.

[17] Biron CA, Nguyen KB, Pien GC, Cousens LP, Salazar-Mather TP (1999). Natural killer cells in antiviral defense: function and regulation by innate cytokines. Annu Rev Immunol.

[18] Guma M, Angulo A, Vilches C, Gomez-Lozano N, Malats N, Lopez-Botet M (2004). Imprint of human cytomegalovirus infection on the NK cell receptor repertoire. Blood..

[19] Redondo-Pachon D, Crespo M, Yelamos J, Muntasell A, Perez-Saez MJ, Perez-Fernandez S (2017). Adaptive NKG2C+ NK cell response and the risk of Cytomegalovirus infection in kidney transplant recipients. J Immunol.

[20] Muntasell A, Lopez-Montanes M, Vera A, Heredia G, Romo N, Penafiel J (2013). NKG2C zygosity influences CD94/NKG2C receptor function and the NK-cell compartment redistribution in response to human cytomegalovirus. Eur J Immunol.

[21] Noble BJ (1982). Clinical applications of perceived exertion. Med Sci Sports Exerc.

[22] Mahler DA, Rosiello RA, Harver A, Lentine T, McGovern JF, Daubenspeck JA (1987). Comparison of clinical dyspnea ratings and psychophysical measurements of respiratory sensation in obstructive airway disease. Am Rev Respir Dis.

[23] Roca J, Sanchis J, Agusti-Vidal A, Segarra F, Navajas D, Rodriguez-Roisin R (1986). Spirometric reference values from a Mediterranean population. Bull Eur Physiopathol Respir.

[24] Roca J, Rodriguez-Roisin R, Cobo E, Burgos F, Perez J, Clausen JL (1990). Single-breath carbon monoxide diffusing capacity prediction equations from a Mediterranean population. Am Rev Respir Dis.

[25] Roca J, Burgos F, Barbera JA, Sunyer J, Rodriguez-Roisin R, Castellsague J (1998). Prediction equations for plethysmographic lung volumes. Respir Med.

[26] Schols AM, Slangen J, Volovics L, Wouters EF (1998). Weight loss is a reversible factor in the prognosis of chronic obstructive pulmonary disease. Am J Respir Crit Care Med.

[27] Crespo M, Yelamos J, Redondo D, Muntasell A, Perez-Saez MJ, Lopez-Montanes M (2015). Circulating NK-cell subsets in renal allograft recipients with anti-HLA donor-specific antibodies. Am J Transplant.

[28] Moraru M, Canizares M, Muntasell A, de Pablo R, Lopez-Botet M, Vilches C (2012). Assessment of copy-number variation in the NKG2C receptor gene in a single-tube and characterization of a reference cell panel, using standard polymerase chain reaction. Tissue Antigens.

[29] Phan MT, Chun S, Kim SH, Ali AK, Lee SH, Kim S (2017). Natural killer cell subsets and receptor expression in peripheral blood mononuclear cells of a healthy Korean population: reference range, influence of age and sex, and correlation between NK cell receptors and cytotoxicity. Hum Immunol.

[30] Robles C, Casabonne D, Benavente Y, Costas L, Gonzalez-Barca E, Aymerich M (2015). Seroreactivity against Merkel cell polyomavirus and other polyomaviruses in chronic lymphocytic leukaemia, the MCC-Spain study. J Gen Virol.

[31] Vivier E, Tomasello E, Baratin M, Walzer T, Ugolini S (2008). Functions of natural killer cells. Nat Immunol.

[32] Holder Kayla A., Grant Michael D. (2019). Human cytomegalovirus IL‐10 augments NK cell cytotoxicity. Journal of Leukocyte Biology.

[33] Lopez-Botet M, Muntasell A, Martinez-Rodriguez JE, Lopez-Montanes M, Costa-Garcia M, Pupuleku A (2016). Development of the adaptive NK cell response to human cytomegalovirus in the context of aging. Mech Ageing Dev.

[34] Costabel U, Maier K, Teschler H, Wang YM (1992). Local immune components in chronic obstructive pulmonary disease. Respir..

[35] Urbanowicz RA, Lamb JR, Todd I, Corne JM, Fairclough LC (2010). Enhanced effector function of cytotoxic cells in the induced sputum of COPD patients. Respir Res.

[36] Tang Y, Li X, Wang M, Zou Q, Zhao S, Sun B (2013). Increased numbers of NK cells, NKT-like cells, and NK inhibitory receptors in peripheral blood of patients with chronic obstructive pulmonary disease. Clin Dev Immunol.

[37] Folli C, Chiappori A, Pellegrini M, Garelli V, Riccio AM, De Ferrari L (2012). COPD treatment: real life and experimental effects on peripheral NK cells, their receptors expression and their IFN-gamma secretion. Pulm Pharmacol Ther.

[38] Prieto A, Reyes E, Bernstein ED, Martinez B, Monserrat J, Izquierdo JL (2001). Defective natural killer and phagocytic activities in chronic obstructive pulmonary disease are restored by glycophosphopeptical (inmunoferon). Am J Respir Crit Care Med.

[39] Urbanowicz RA, Lamb JR, Todd I, Corne JM, Fairclough LC (2009). Altered effector function of peripheral cytotoxic cells in COPD. Respir Res.

[40] Wang J, Urbanowicz RA, Tighe PJ, Todd I, Corne JM, Fairclough LC (2013). Differential activation of killer cells in the circulation and the lung: a study of current smoking status and chronic obstructive pulmonary disease (COPD). PLoS One.

[41] Morissette MC, Parent J, Milot J (2007). Perforin, granzyme B, and FasL expression by peripheral blood T lymphocytes in emphysema. Respir Res.

[42] Finch DK, Stolberg VR, Ferguson J, Alikaj H, Kady MR, Richmond BW (2018). Lung dendritic cells drive natural killer cytotoxicity in chronic obstructive pulmonary disease via IL-15Ralpha. Am J Respir Crit Care Med.

[43] Freeman CM, Stolberg VR, Crudgington S, Martinez FJ, Han MK, Chensue SW (2014). Human CD56+ cytotoxic lung lymphocytes kill autologous lung cells in chronic obstructive pulmonary disease. PLoS One.

[44] Boyton RJ, Smith J, Ward R, Jones M, Ozerovitch L, Wilson R (2006). HLA-C and killer cell immunoglobulin-like receptor genes in idiopathic bronchiectasis. Am J Respir Crit Care Med.

[45] Boyton RJ, Altmann DM (2007). Natural killer cells, killer immunoglobulin-like receptors and human leucocyte antigen class I in disease. Clin Exp Immunol.

[46] Boyton RJ (2009). Regulation of immunity in bronchiectasis. Med Mycol.

[47] Motz GT, Eppert BL, Wortham BW, Amos-Kroohs RM, Flury JL, Wesselkamper SC (2010). Chronic cigarette smoke exposure primes NK cell activation in a mouse model of chronic obstructive pulmonary disease. J Immunol.

[48] Morrow JD, Qiu W, Chhabra D, Rennard SI, Belloni P, Belousov A (2015). Identifying a gene expression signature of frequent COPD exacerbations in peripheral blood using network methods. BMC Med Genet.

[49] Rijavec M, Volarevic S, Osolnik K, Kosnik M, Korosec P (2011). Natural killer T cells in pulmonary disorders. Respir Med.

[50] Hodge G, Holmes M, Jersmann H, Reynolds PN, Hodge S (2014). Targeting peripheral blood pro-inflammatory cytotoxic lymphocytes by inhibiting CD137 expression: novel potential treatment for COPD. BMC Pulm Med.

[51] Roos-Engstrand E, Pourazar J, Behndig AF, Blomberg A, Bucht A (2010). Cytotoxic T cells expressing the co-stimulatory receptor NKG2 D are increased in cigarette smoking and COPD. Respir Res.

[52] Borchers MT, Wesselkamper SC, Curull V, Ramirez-Sarmiento A, Sanchez-Font A, Garcia-Aymerich J (2009). Sustained CTL activation by murine pulmonary epithelial cells promotes the development of COPD-like disease. J Clin Invest.

[53] Wortham BW, Eppert BL, Motz GT, Flury JL, Orozco-Levi M, Hoebe K (2012). NKG2D mediates NK cell hyperresponsiveness and influenza-induced pathologies in a mouse model of chronic obstructive pulmonary disease. J Immunol.

[54] Soderberg-Naucler C, Streblow DN, Fish KN, Allan-Yorke J, Smith PP, Nelson JA (2001). Reactivation of latent human cytomegalovirus in CD14(+) monocytes is differentiation dependent. J Virol.

[55] Soderberg-Naucler C, Fish KN, Nelson JA (1997). Interferon-gamma and tumor necrosis factor-alpha specifically induce formation of cytomegalovirus-permissive monocyte-derived macrophages that are refractory to the antiviral activity of these cytokines. J Clin Invest.

[56] Forte E, Swaminathan S, Schroeder MW, Kim JY, Terhune SS, Hummel M. Tumor Necrosis Factor Alpha Induces Reactivation of Human Cytomegalovirus Independently of Myeloid Cell Differentiation following Posttranscriptional Establishment of Latency. MBio. 2018;9(5). 10.1128/mBio.01560-18 PMC6134100 30206173

